# Acceptability and Satisfaction of Contraceptive Vaginal Rings in Clinical Studies: A Systematic Review and Narrative Synthesis

**DOI:** 10.3389/fgwh.2021.799963

**Published:** 2021-12-14

**Authors:** Thérèse Delvaux, Vicky Jespers, Lenka Benova, Janneke van de Wijgert

**Affiliations:** ^1^Institute of Tropical Medicine, Antwerp, Belgium; ^2^Belgian Health Care Knowledge Centre, Brussels, Belgium; ^3^Julius Center for Health Sciences and Primary Care, University Medical Center Utrecht, Utrecht University, Utrecht, Netherlands

**Keywords:** contraceptive vaginal ring, hormonal contraception, acceptability, satisfaction, sexual satisfaction

## Abstract

**Introduction:** Acceptability of and satisfaction with contraceptive methods are paramount for uptake and continuation. In the current context of multipurpose prevention of pregnancy and sexually transmitted diseases/HIV development, it is critical to have a better understanding of acceptability of and satisfaction with the contraceptive vaginal ring (CVR) including sexual satisfaction. The objective of this study was to review the evidence about acceptability of CVRs and general and sexual satisfaction of users.

**Methods:** We searched PubMed, CINAHL, and Web of Science (until December 31, 2020) and selected original studies documenting actual use of hormonal CVR and explicitly addressing any of the 3 outcomes.

**Results:** Of a total of 1,129 records screened, 46 studies were included. Most studies (*n* = 43, 93%) were prospective, conducted in high-income settings (*n* = 35), and reported on NuvaRing^®^ use (*n* = 31). Overall, 27 (59%) studies included a comparison group, 38 (82%) studies used exclusively quantitative questionnaires, with qualitative only (*n* = 4, 9%), or mixed methods (*n* = 4, 9%) studies being less common. Ease of CVR insertion/removal/reinsertion was high in all the settings and improved with time of use, with qualitative studies supporting these findings. When mentioned, ring-related events were associated with discontinuation, and results on continuation of use were mixed. Among NuvaRing^®^ studies, general satisfaction (being satisfied or very satisfied) was between 80 and 90% and tended to mirror continuation. Sexual satisfaction was less commonly reported and results were mixed. Overall, limited information was provided on actual CVR experiences of women (and men) and cultural norms that may affect sexuality and CVR use.

**Conclusion:** Positive aspects of acceptability of and satisfaction with CVRs were reported, but ring-related events and factors, which may affect long-term CVR use, deserve further study. More information is needed on actual experiences of women using CVRs, relationship aspects, male partner opinions, and contextual norms to better understand the acceptability of and satisfaction with CVRs.

## Introduction

Contraceptive vaginal rings (CVRs) have been developed since 1970 and 3 CVRs are currently available: the etonogestrel and ethinyl estradiol ring (marketed as NuvaRing^®^), the progesterone ring for breastfeeding women (Progering^®^), and the recently approved segesterone acetate (previously called Nestorone) and ethinyl estradiol ring (Annovera™) (1, 2). Advantages of CVRs are multiple: they are user-initiated and controlled, independent of sexual acts, and can provide long-term effective protection (1). Moreover, vaginal rings could be designed to include several active ingredients that provide prevention for HIV, other sexually transmitted infections (STIs), and pregnancy (3).

Acceptability of and satisfaction with contraceptive methods impact uptake, adherence, and continuation and, therefore, contribute significantly to contraceptive effectiveness (4). In clinical studies, acceptability of contraceptive methods is often documented through the effect of the product on bleeding patterns/cycle control, its side effects, and the duration of use. Satisfaction tends to reflect the perceptions of the product of user and is assessed quantitatively through levels of satisfaction during actual use and/or indirectly assessed through willingness to use in the future or recommend the method (1). Both the concept of acceptability and satisfaction are in fact intertwined as illustrated by validated quantitative tools in which overall satisfaction is considered a dimension of acceptability (5, 6). Moreover, given vaginal administration, CVRs may affect sexual relationships. To this end, sexual satisfaction with CVR has been studied more specifically using sexual function assessment tools such as the Female Sexual Function Index (7). In reality, acceptability and satisfaction are complex concepts that are influenced by physical, behavioral, physiological, interpersonal, and structural factors. Recent studies documenting the effectiveness of vaginal products and devices in the field of HIV prevention have confirmed the key contribution of acceptability to adherence and theoretical frameworks presenting pathways from various acceptability dimensions toward satisfaction and then to adherence have been developed to aid further inquiry (8).

Given the current focus and importance of multipurpose technology for prevention of pregnancy and STIs/HIV, it is critical to have a better understanding of what is commonly considered as acceptability and satisfaction of CVR and main reported results with respect to these outcomes including sexual satisfaction. The objectives of this study were to review the overall evidence of acceptability of CVRs and general and sexual satisfaction of users.

## Materials and Methods

### Protocol and Registration

This study protocol was registered on the international prospective register of systematic reviews (PROSPERO) (CRD42017079157).

### Literature Search

Databases searched were PubMed, cumulative index to nursing and allied health literature (CINAHL), and Web of Science with a cutoff date of December 31, 2020. The main search terms were “contraceptive vaginal ring” and “acceptability” or “satisfaction” or “sexual satisfaction” and synonyms of each of these terms were also included. Additional search terms included “qualitative methods,” “mixed methods,” and “trials.” The search strategies were adjusted according to the specifications of each database. Additional relevant publications from other sources (reference lists) were also included (Supplementary Material S1_Search strategy). The Preferred Reporting Items for Systematic Reviews and Meta-Analyses (PRISMA) framework guidelines, flow diagram, and checklist were utilized to undertake this study.

### Selection Criteria

Studies were eligible if they included *actual CVR use* by healthy women of reproductive age (15–49 years) and *explicitly* addressed acceptability, satisfaction, and/or sexual satisfaction. We did not use specific definitions of acceptability and satisfaction because we wanted to learn which definitions or concepts the various authors had used. Similarly, we did not select studies based on study methods used, but excluded reviews and opinion papers or commentaries, validation studies, studies that evaluated non-contraceptive vaginal ring use (such as rings for hormonal replacement therapy), or assessed acceptability or willingness to use hypothetically in the absence of actual user experiences. Studies that only enrolled women with a specific health condition (such as diabetes) and full texts in languages other than English, French, Dutch, Spanish, or Italian were also excluded (*n* = 3). In case of multiple articles presenting data from the same study with the same outcomes of interest, only the primary paper was included in this study (Excluded studies in Supplementary Material S2).

### Study Selection

Each title and abstract were screened by two independent reviewers (TD and VJ) using the inclusion criteria described above. Full texts of all the papers selected in title and abstract screening were checked by both the reviewers before inclusion and any discrepancies were discussed until consensus was reached.

### Study Quality Assessment and Data Synthesis

A standardized pretested form was used by TD to extract data from full texts on study characteristics: author names, year of publication, journal, study setting, study design, ring use and comparison group(s) (if any), research methods used and main findings related to acceptability, overall satisfaction and sexual satisfaction. Data on sample size, randomization process, and presence of a control group related to methodological quality assessment were also extracted, but were not considered a core component of this study, as we wanted to provide an overview of methods used to document acceptability and satisfaction.

### Patient and Public Involvement

No patient or public involvement took place in the design or conduct of this systematic review, which included 46 papers from many countries worldwide.

## Results

**Figure 1:**
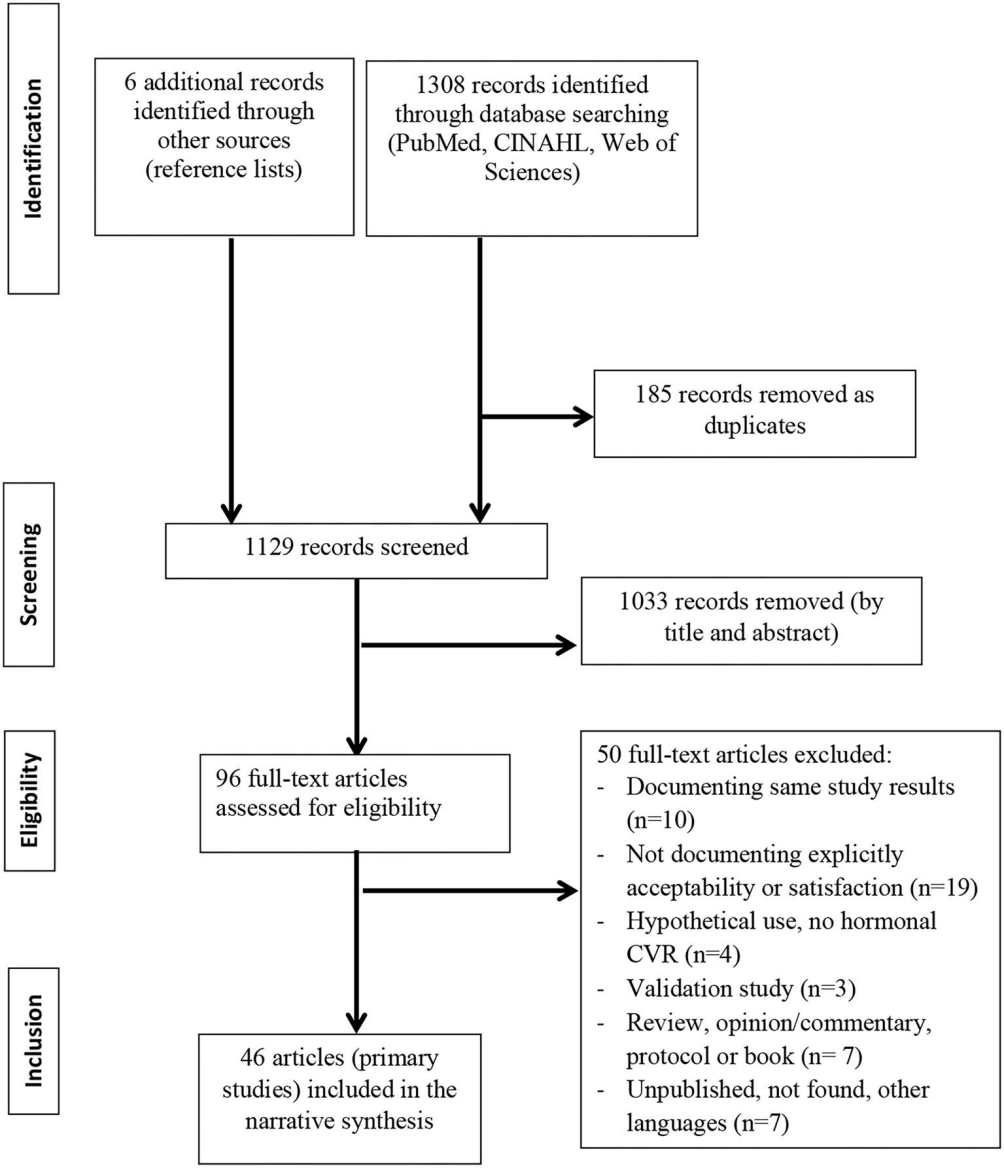
The Preferred Reporting Items for Systematic Reviews and Meta-Analyses (PRISMA) flowchart of the studies selection process for the review on contraceptive vaginal rings, acceptability, and general and sexual satisfaction.

Of 1,308 publications that were identified through database searching, after removal of duplicates, 1,129 titles/abstracts and 96 full texts were reviewed and 46 articles (primary studies) were included (Figure 1).

### Studies Design, Methods, Characteristics, and Settings

A total of 19 studies (41%) were randomized clinical trials (9–27), 24 studies were prospective non-randomized studies (28–51), and 3 studies were cross-sectional studies (52–54) (Table 1). A total of 27 studies (59%) used a controlled design, comparing CVR users to users of other hormonal methods [such as a combined oral contraceptive (COC) pill or patch] or to non-hormonal contraceptive methods (such as the copper intrauterine device) or comparing users of CVRs containing different hormonal dosages or the same CVR for different durations. The remaining 19 studies did not include a comparison group (Table 1).

**Table 1:** Types of study design, methods, participants, and study duration.

**Type of study** ***N*** **(%)**		**Comparison group** ***N*** **(%, per study type)**	**Quantitative methods** **structured questionnaire**	**Qualitative methods** ^*^ **Semi-structured, IDI, FGD**	**Study participants** ***N*** **Range**	**Study duration** ***N*** **months Range**
Prospective randomized (9–27)	**19 (41)**	19 (100)	19 (100)	2 (11)	14–983	1–13 months
Prospective non randomized (28–51)	**24 (53)**	7 (30)	21 (88)	5 (21)	27–5823	2–24 months
Cross sectional (52–54)	**3 (7)**	1 (33)	2 (67)	1 (33)	32–26,250	-
**Total**	**46 (100)**	**27 (59)**	**42 (91)**	**8 (17)**	**14**–**26,250**	-

* *Four studies used both the quantitative and qualitative methods (mixed methods). IDI, in-depth interview; FGD, focus group discussion. Total rows and columns are presented in bold*.

Most studies (42/46, 91%) used quantitative structured questionnaires and 8 (17%) studies used qualitative semi-structured or in-depth interviews (IDIs) and/or focus group discussions (FGDs) (Table 1). Overall, 38 (82%) studies used exclusively quantitative structured questionnaires (25, 26, 30, 36, 41, 44, 49, 52), while 4 (9%) studies used only qualitative methods and 4 (9%) studies used both the quantitative and qualitative methods (25, 26, 36, 49). The number of participants in the prospective studies ranged from 14 to 5,823, with an average 50 to 200 participants. In total, 1 cross-sectional study was larger with up to 26,250 participants. The duration of use in the prospective studies ranged from 1 to 24 menstrual cycles, with about a third (*n* = 13) covering 12 cycles or more, another third (*n* = 13) covering 6 to 12 menstrual cycles, and the remaining studies covering 3 menstrual cycles or fewer (Table 1).

Studies were performed in 1 or more high-income settings, i.e., countries of Europe, USA, Canada, or Australia (*n* = 35/46 studies, 76%); Latin America (*n* = 5); Israel (*n* = 2); Asia (*n* = 5, of which 3 were in India); and Africa [*n* = 5, including 1 study each in Rwanda, Kenya, South Africa, and 2 studies in several (mostly sub-Saharan) African countries] (Table 2).

**Table 2:** Study settings and types of contraceptive vaginal rings.

**Types of contraceptive vaginal ring**	***Study settings***					
	***Europe, USA, Canada, Australia $*** ***N***	***Latin America*** ***N***	***Israel*** ***N***	***Asia §*** ***N***	***Africa*** ***N***	**All studies** ***N*** **(%)**
NuvaRing$$	24	-	2	3	3	31^*^ **(67)**
Annovera™	2	2			1	2^*^ **(4)**
Progering^®^	-	1		-	-	**2 (4)**
Levonorgestrel	3			2	1	4^*^ **(9)**
Levonorgestrel/Estradiol	1	2			-	**3 (7)**
Other CVR	4	-			-	**4 (9)**
**Total, N (%)**	**35 (76)**	**5 (11)**	**2 (4)**	**5 (11)**	**5 (9)**	**46** ^*^ **(100)**

* *High-income settings: Europe, USA, Canada, Australia, and Israel. “All studies” column values are less than the sum of all column values, as some studies include several settings;*

$: *Any of these settings or several of them;*

§: *Including 3 studies in India;*

$$: *including 3 study with a new etonogestrel/ethinyl estradiol ring (Kirkos^®^). Total rows and columns are presented in bold*.

NuvaRing^®^ was the most studied CVR (31/46 studies, including 1 recent study testing a new etonogestrel/ethinyl estradiol—Kirkos^®^—against NuvaRing^®^), while 2 studies evaluated use of the Annovera™ ring and 2 other studies evaluated use of the Progering^®^ among breastfeeding women. The remaining 11 studies investigated CVRs containing levonorgestrel (LNG) alone, combined with ethinyl estradiol or other progesterone regimens. These CVRs were not further developed and did not make it to the market (Table 2).

### Main Findings on Acceptability, Satisfaction, and Sexual Satisfaction

Overall definitions of acceptability and satisfaction or how these outcomes were described, varied across studies, over time and according to the type of CVR. Therefore, we will present main results on these outcomes by type of CVR.

#### NuvaRing^®^

Studies documenting acceptability and/ or satisfaction of NuvaRing^®^ commonly used structured questionnaires assessing the following similar dimensions: clarity of instructions; ease of use (including to insert/remove the ring); ease of package use; compliance or adherence (including removals and spontaneous expulsions); cycle-related characteristics (menstrual changes or pain); sexual comfort (whether the ring was felt by the woman or the male partner or whether the partner objected to the ring, without investigating sexual frequency, pleasure, or satisfaction); and overall satisfaction. These seven dimensions were included in a validated 21-item questionnaire by Novak et al. (5) and subsequently used in other studies (21, 31–33, 37, 38, 48). The IUD intra uterine device (ORTHO-BC-SAT) satisfaction questionnaire related to the use of hormonal contraception in general and including 8 dimensions similar to Novack et al. questionnaire that was used in 1 CVR study (19). Over 80% of NuvaRing^®^ users in all the studies using the seven/eight dimensions showed that CVR instructions and packaging were clear and that the ring was easy to insert and remove (Table 3). In Kenya, a qualitative study showed that unease with vaginal insertion and ring placement issues (slippage and expulsion) created initial challenges requiring clinician assistance and practice for some participants (30). Similarly, an in-depth discussion with users in Rwanda showed that initial worries with respect to CVR insertion reduced over time with actual ring use and ring insertions and removals were, henceforth, described as easy (26). On the other hand, 2 studies reported that the previous use of tampons did not seem to influence satisfaction or successful ring use of CVR (14, 52). A number of studies reported spontaneous expulsions rates ranging from less than 2% (15, 17) to 5–20% (21, 36, 46, 51). A study in Switzerland showed that 17.5% of adverse events were ring related such as feeling the ring, vaginal discomfort, and vaginal expulsions (31). In a study in the Netherlands (38), women who felt the ring were more likely to remove it (sometimes, regularly, and always) during intercourse compared to those who did not feel it (22 vs. 6%) (Table 3). An in-depth study among adolescents in the US using NuvaRing^®^ revealed that 5 of 32 participants discontinued because of ring-related events (52). In Kenya, minor side effects were described and concerns centered on ring efficacy, negative effect on a sexual desire of woman, future fertility issues, and non-suppression of menstruation, which were favored by most participants (30).

**Table 3:** Study characteristics, type of CVR and study, participants, comparison group, outcome(s), methods used, and main results, presented by type of CVR then chronologically.

**Authors, Public. year, Setting**	**Type of CVR**	**Type Study**	**Total sample**	**CVR users**	**Comparison**	**Follow-up**	**Outcomes**	**Methods**	**Main results: acceptability, satisfaction, sexual satisfaction**
Gill et al., 2020, South Africa (19)	NuvaRing^®^	PR	150	116	3 arms: Injectable, COC; Cross-over	4 cycles	Acceptability satisfaction	Quantitative Questionnaire: including self-administered ORTHO birth control satisfaction (BC-SAT)	More NuvaRing^®^ users (24/116; 20.7%) significantly requested to change to another contraceptive option compared to injection (1/73; 1.4% *p* = 0.0002) and COC users (4/49; 8% *p* = 0.074). Significantly more injection users (77/80; 96.3%) thought this delivery mode was convenient to use compared to NuvaRing^®^ (74/89; 83.1%; *p* = 0.0409) or COC (38/50; 76.0%; *p* = 0.0034). Overall, the preferred contraceptive choice was injection, followed by the ring and lastly the pill.
Caruso et al., 2019 (20)	Kirkos^®^ / NuvaRing^®^	PR	58	29 (Kirkos^®^)	29 (NuvaRing^®^)	6 cycles	Sexual satisfaction	Quantitative: Questionnaire; Diary; Female Sexual Function Index (FSFI) self-administered; SF-36 and Female Sexual Distress Scale (FSDS); Quality sexual life; 0–100 Visual Analog Scale (pain)	Improvement of sexual function scores among women using Kirkos^®^ vs. NuvaRing^®^ both at the 1st (FSFI, *p* < 0.009; FSDS, *p* < 0.001) and at the 2nd (FSFI, *p* < 0.001; FSDS, *p* < 0.002) follow-up. QoL of Kirkos^®^ users improved at the 1st follow-up (*p* < 0.05) and 2nd (*p* < 0.01) follow-up. NuvaRing^®^ users improved their QoL at the 2nd follow-up (*p* < 0.01).
Kestelyn et al., 2018 Rwanda (26)	NuvaRing^®^	PR	130	120, 10 males partners	2 arms intermittent/ continuous CVR use	3 cycles	Acceptability satisfaction sexual satisfaction	Mixed- methods: Questionnaire, In-depth Interviews; focus group discussion, diary, ballot box (self-administered anonymous), observation	Initial worries regarding CVR reduced over time with actual ring use; ring insertions and removals described as easy. Most women did not feel the ring during daily activities, appreciated the lack of perceived negative side effects. Sexual comfort (increased lubrication) played a significant role in ring acceptability of the participants and their partners. Rwandan cultural norms around sexuality positively influenced the acceptance of the NuvaRing^®^ Overall satisfaction was high.
Guida et al., 2017, Italy (48)	NuvaRing^®^	CS	556	76	5 groups (COC, implant; no contraception)	-	Sexual satisfaction	Quantitative: McCoy Female Sexuality Questionnaire (MFSQ)+ Ultrasound dorsal clitoral artery	Statistically significant lower McCoy value in CVR group vs. the implant group.
McLellan-Lemal et al., 2017, Kenya (30)	NuvaRing^®^	PNR	44	24, 20 males partners	-		Acceptability	Qualitative component in prospective clinical study: Ethnographic research- In-depth interviews women & male partners	Unease with vaginal insertion as well as potential slippage or expulsion created initial challenges requiring clinician assistance and practice for some participants. Minor side-effects were described. Awareness of the multiple contexts in ring users' experience may inform the development, education, and promotion approaches for future ARV rings. Experiences with CVR reflected a broader Family Planning (FP) paradigm (i.e. ring efficacy & future fertility issues, “feeling free” to stop, lack of side effects including negative effect on a woman's sexual desire).
Dam et al., 2015, India (45)	NuvaRing^®^	PNR	45	45	-	3 cycles	Acceptability satisfaction sexual satisfaction	Quantitative: Questionnaire	96% women were satisfied with the ring usage; 97% would recommend it to others; Sexual comfort: 30% women could feel the ring, 18% partners felt the ring during intercourse whereas in 21% cases partner minded that women were using the ring.
Guida et al., 2014, Italy (49)	NuvaRing^®^	PNR	556	76	60 (Patch); 128, 88, 64 (COC); 140 (no hormonal)	2 extended cycles	Sexual satisfaction	Mixed-methods: Quantitative & Semi structured Interview (Sexual life IRSF); 1–100 Visual Scale	Significant reduction in anxiousness relating to sexual activity, in all groups using contraception compared to controls.
Battaglia et al., 2014, Italy (10)	NuvaRing^®^	PR	43	21	22 (COC)	6 cycles	Sexual satisfaction	Quantitative: Questionnaire; McCoy Female Sexuality Questionnaire (MFSQ); self-administered Beck's Depression Inventory questionnaire (BDI); + Ultrasound clitoridal artery & Hormonal	Significant decrease of the two-factor Italian MFSQ score for COC & CVR users, which was more marked in OC users; Frequency of sexual intercourse and orgasm was reduced only by the use of OC.
Caruso et al., 2014, Italy (40)	NuvaRing^®^	PNR	52	52	-	2 extended cycles	Sexual satisfaction	Quantitative: Questionnaire; diary; Self-administered Female Sexual Function Index (FSFI); SF-36 and Female Sexual Distress Scale (FSDS); Quality sexual life	Improvement of FSFI and FSDS scores obtained at the first and second follow-up appointments vs. baseline scores (*p* < 0.05). QoL measures of body pain, general health and emotional role improved at the first follow-up visit (*p* < 0.05); at the second one, all variables showed improvement (*p* < 0.05).
Pandit et al., 2014, India (34)	NuvaRing^®^	PNR	252	252	-	3 cycles	satisfaction	Quantitative: Questionnaire; (5 items satisfaction Likert Scale)	92% agreed that instructions for CVR easy to follow; Satisfaction: 94% very satisfied; 93% would recommend others.
Soni et al., 2013, India (43)	NuvaRing^®^	PNR	184	184	-	13 cycles	Acceptability	Quantitative: Questionnaire and diary	Compliance was good (99%); 0.16% incidence of intermenstrual bleeding and 2% incidence of early withdrawal bleeding; “ the ring is highly acceptable to users”.
Elaut et al., 2012, Belgium (22)	NuvaRing^®^	PR	55	55	Consecutive use (CVR, COC, POP)	3 cycles each method	Sexual satisfaction	Quantitative: including self-administered questionnaire (on relationship and psychosexual measures	Sexual desire higher among ring users (*P* < 0.0001); woman's mood positively impacted.
Peipert et al., 2011, USA (35)	NuvaRing^®^	PNR	4,167	431	*N* = 1890 LNG IUD; 431 IUD; 552 implant; 478 COC; 313 DMPA; 99 Patch	12 cycles	Acceptability satisfaction	Quantitative: Questionnaire (phone survey)	At 12 months continuation rates were at 86% (for long-acting reversible contraception (IUD and implant) users), 57% (DMPA),55% (Ocs), 54% (CVR), and 49% for patch users. Satisfaction mirrored continuation.
Gilliam et al., 2010, USA (23)	NuvaRing^®^	PR	273	136	137 (COC)	3 cycles	Acceptability	Quantitative: online questionnaire survey and daily internet-based diaries	At 6 months, similar proportions (26 and 29% of CVR and COC users, respectively) had continued their assigned study method (*P* = 0.61).
Gracia et al., 2010, Italy (24)	NuvaRing^®^	PR	499	249	250 (Patch)	3 cycles	Sexual satisfaction	Quantitative: Questionnaire; Self administered Female Sexual Function Index (FSFI)	Mean scores at endpoint in subjects with a male partner were significantly lower in the CVR group (27.4 with contraceptive ring vs. 29.2 with contraceptive patch.
Merki-Feld, 2010, Switzerland (32)	NuvaRing^®^	PNR	1,053	1,053	-	4 cycles	satisfaction	Quantitative: Questionnaire	Women were satisfied with changes in weight (92%), cycle control (94%) and Post Menstrual Syndrome (86%). Cycle regularity significantly imporved among starters compared to switchers. Adverse events were reported for 17.5% of women and were most frequently ring-related (such as feeling the ring *in situ*, vaginal discomfort, ring expulsion).
Lete et al., 2008, Spain (54)	NuvaRing^®^	CS	26,250	23%	77% other methods	-	Acceptability	Quantitative: Self-administered questionnaire; 1 to 5 or six-point Visual Analog Scale	A similar percentage of women in the pill and skin patch groups changed to CVR (31.6 and 32.9%, respectively), whereas among CVR users only 1% changed to the pill and 3% to the skin patch.
Creinin et al., 2008, USA (21)	NuvaRing^®^	PR	500	249	251 (Patch)	4 cycles	Acceptability	Quantitative: Questionnaire and Visual Analog Scale (VAS) for acceptability	More CVR users (71.0%) planned to continue their method after the study than Patch users (26.5%) (*P* < 0.001). Ring users preferred the ring to the combined OC (*p* = *0*.001), and patch users preferred the combined OC to the patch (*p* = 0.001).
Epstein et al.,2008, USA (52)	NuvaRing^®^	CS	32	-	-	-	Acceptability sexual satisfaction	Qualitative: In- Depth Interviews (*n* = 32 adolescents)	An adjustment period (to become more comfortable using the ring) was reported by most participants. In total, 5 of 32 participants (16%) discontinued ring use, 3 of them because the ring could be felt in the vagina during intercourse, or always; 1 because she disliked touching her vagina. Participants said it was important to warn partners about the ring before sexual contact, not to “surprise” them if they felt the ring inside the vagina. In total, 4 of 32 participants reported removing the ring during sex (felt uncomfortable). Prior use of tampons did not seem to increase successful ring use.
Brucker et al., 2008, Germany (28)	NuvaRing^®^	PNR	5,823	5,823	-	8 cycles	Acceptability satisfaction sexual satisfaction	Quantitative: Questionnaire	CVR well tolerated (Bleeding patterns, blood pressure), Most women expressed their satisfaction with CVR; 82% were “very satisfied/satisfied”, 72% planned to continue using it and 82% would recommend it to others. More than 90% of women found NuvaRing1 “without problems/easy” to insert and to remove, and more than 80% of the women and their partners were not disturbed by its presence during intercourse.
Stewart et al., 2007, USA (18)	NuvaRing^®^	PR	130	130	130 (COC) Consecutive use	3 cycles each	Acceptability	Quantitative: Questionnaire; Computer-assisted self-interviewing software	Overall approval higher among CVR users i.e. liked using method (*P* = 0.015), would recommend it to friends (*P* = 0.012), and not as hard to remember to use method correctly (*P* ≤ 0.000). Participants were less worried about health risks while using the ring (*P* = 0.006), but reported that the ring was more likely to interfere with sex than the pill (*P* ≤ 0.001) and that sex partners liked the pill (*P* = 0.034).
Merki -Feld & Hund, 2007, Switzerland (31)	NuvaRing^®^	PNR	2,642	2,642	-	3–7 cycles	Acceptability satisfaction	Quantitative: Questionnaire	Overall 85% were satisfied/very satisfied, 58% were very satisfied with CVR use. 89% would recommend to others and 74% wished to continue. Satisfaction improved with duration of treatment.
Fine et al., 2007, USA (48)	NuvaRing^®^	PNR	81	81	-	3 cycles	Acceptability satisfaction	Quantitative: Questionnaire	Overall satisfaction and acceptability of CVR among postabortion patients was high. 89 % participants elected to continue the CVR, nearly all would recommend this method to a friend.
Ahrendt et al., 2006, 10 European countries (9)	NuvaRing^®^	PR	983	499	484 (COC)	13 cycles	Acceptability	Quantitative: Questionnaire and diary	The vast majority of women found CVR easy to insert (96%) and remove (97%). Non significant difference in continuation with CVR (71%) vs. CoC (75%). Satisfaction was high (84% CVR vs. 87% COC); recommending to others (87% NuvaRing; 92% COC).
Roumen et al., 2006, The Netherlands (38)	NuvaRing^®^	PNR	1,130	1,130	-	3 cycles	Acceptability satisfaction	Quantitative: including self-administered online questionnaire	94% found CVR easy to insert and 97 easy to remove. 87% of women and 67% of partners never felt the ring during intercourse. (Very) satisfied users varied from 34% to 72%; (Very) dissatisfied varied from 44 to 16% over 3 cycles.
Sabatini & Cagiano, 2006 Italy (13)	NuvaRing^®^	PR	280	94	94,92, (COC: group 1 20μEE; group 2: 15μEE)	12 cycles	Sexual satisfaction	Quantitative: Questionnaire; Irritability, depression side effects 3-point scale; Diary	Sexual desire was increased or unchanged in 68% (COC group1), 59% (COC group2) and 91% (CVR group) of the cases. Better results related to desire and sexual satisfaction were obtained by CVR users. The analysis of adverse events revealed two crucial points for acceptability, compliance and continuation: poor cycle control and disturbance of sexual intercourse due to vaginal dryness and loss of desire.
Schafer et al., 2006, USA (14)	NuvaRing^®^	PR	201	101	100 (COC)	3 cycles	Acceptability satisfaction	Quantitative: including Self-administered questionnaire (on sexual story)	Higher satisfaction among CVR users (61%) vs. pill users (34%) (*p* = 0.003). No association between satisfaction at 3 months and report of previous genital touching (tampon etc.) at baseline.
Guida et al., 2005, Italy (25)	NuvaRing^®^	PR	116	26	25 (COC) + 23, 25 (implant, non-hormonal) non-randomized	6 cycles	Sexual satisfaction	Mixed-methods: Quantitative; & Semi structured Interview: Sexual life and Interviewer Rating Sexual Function (IRSF); (0-100) Visual Analog Scale	CVR seems to implement a further positive effect on the psychological aspect of both women and their partners, which is evident from an improved complicity and sexual satisfaction.
Miller et al., 2005, 4 countries Europe, USA (11)	NuvaRing^®^	PR	429	429	4 arms extended use (with increased duration)	12 cycles	Acceptability satisfaction	Quantitative: including Self-administered Questionnaire	One year treatment completion rates were higher with shorter regimens and ranged from 77% to 59%.The highest satisfaction was reported for the shorter (91%) and the lowest for the longest (77%) regimens.
Novak 2003, Europe, Israel, USA, Canada (33)	NuvaRing^®^	PNR	2,393	2,393	-	13 cycles	Acceptability satisfaction	Quantitative: including self-administered Questionnaire (21-item acceptability	85% and 90-of women were satisfied or very satisfied with the ring and would recommend the ring to others, respectively, increasing to 96 and 97%, respectively, for those who completed the studies. Overall 15% women and 30% partners felt the ring during intercourse (6% partners objected to CVR use).
Roumen et al., 2001, 11 European countries, Israel (37)	NuvaRing^®^	PNR	1,145	1,145	-	13 cycles	Acceptability satisfaction	Quantitative: including 21-item self-administered questionnaire; Diary	96 and 98% women were satisfied and would recommend the method to others (59–67% among women who discontinued, respectively).
Merkatz et al., 2014, Latin America, USA, Europe, Australia (27)	Annovera™	PR	1,036	1,036	Several arms/ different dosages	13 cycles	Acceptability satisfaction sexual satisfaction	Quantitative: Questionnaire (acceptability study in a clinical trial)	Satisfaction was high (89%) and related to higher method adherence [OR, 2.6 (1.3, 5.2)] and continuation [OR, 5.5 (3.5, 8.4)]. Attributes of CVR use representing items from the four domains - finding it easy to remove, not complaining of side effects, not feeling the CVR while wearing it and experiencing no change or an increase in sexual pleasure and/or frequency -were associated with higher odds of satisfaction.
Sivin et al., 2005, Latin Am, USA, Europe (15)	Annovera™	PR	150	150	3 arms different dosages	13 cycles	Acceptability	Quantitative: including self-administered Questionnaire	Overall one-year continuation rates were at 73%. Medical conditions, mainly vaginal problems, personal reasons and device loss or repeated expulsion were the principal reasons given for study discontinuation. Clinical performance and adverse event profiles indicate that each of these 1-year NES/EE rings, used on a 21-day-in and 7-day-out regimen, provided women effective, acceptable and safe long-acting contraception under their own control.
RamaRao et al. 2015, Kenya, Nigeria, Senegal (36)	Progering^®^	PNR	384	174	174 (non CVR users)	2 cycles of 3 months	Acceptability	Mixed-methods: Questionnaire (*n* = 174); In-depth interviews (*n* = 15)	A majority reported the ring was easy to insert/remove/ reinsert at baseline. Perceptions of the ring's size or texture were of more importance than its color at baseline. However perceptions of all these physical aspects became more positive from the time the ring was first seen to the time it was used and there were no significant differences in perception on these 3 aspects between women who had used 2 rings and those who used one. Data indicate that the PVR has limited to no effect on sexual behavior in the post-partum period.
Sanchez et al., 1997, Chile (41)	Progering^®^	PNR	78	63	15 (IUD)	3–14 months	Acceptability	Qualitative methods only: Semi-structured interviews and focus groups discussions; (Acceptability study of a phase III trial)	Most women who used the ring found it highly acceptable and mentioned the following advantages: comfort, efficacy, ease of insertion and removal, user's control, safety, no negative effect on sex life, and prolonged amenorrhoea. Some women disliked these same characteristics or had fears regarding them, and a few women had negative experiences such as excessive vaginal discharge or frequent expulsion
Koetsawong et al., 1990, 13 countries in Asia, Africa, Latin America, Europe (51)	Levonorgestrel (20μ/day)	PNR	1,005	1,005	-	3 cycles	Acceptability	Quantitative: Questionnaire and diary (bleeding pattern)	The principal reasons for discontinuation were menstrual disturbances (17% at 1 year), vaginal symptoms (6.0%) and single or repeated expulsion of the ring (7%).
Buckshee et al., 1990, India (29)	Levonorgestrel (20μ/day)	PNR	96	50 baseline 46 FU	-	12 & 24 cycles	Acceptability	Quantitative: Questionnaire and diary	Follow-up study revealed users to be happier with the ring than with any other method and no spouse complained of feeling the ring during coitus
Sahota et al., 1999, UK (39)	Levonorgestrel (20μg/day)	PNR	1,710	1,710	-	24 cycles	Acceptability	Quantitative: Questionnaire	435/1511 (29%) experienced at least 1 involuntary expulsion; 1-year discontinuation rate was 56% and the 2-year rate was 85%. Over 60% of users found the method to be acceptable at 12 months.
Elder et al., 1991, UK (46)	Levonorgestrel (20μg/day)	PNR	150	150	-	12 cycles	Acceptability	Quantitative: Questionnaire and diary	Menstrual disturbance, vaginal problems (discharge, symptoms) and involuntary expulsion resulted in discontinuation rates of 8.9, 8.4 and 1.6 per 100 woman-years, respectively.
Spencer et al., 1986, UK (44)	Levonorgestrel/ Estradiol	PNR	27	27	-	12 months	Acceptability	Qualitative: In depth Interviews before and during the WHO clinical trial	7/27 women discontinued after 1 year (4, for related CVR reasons); positive features of CVR were that one can forget about it & less deleterious effects on health.
Hardy et al.,1983, Brazil, Dom. Rep. (50)	Levonorgestrel/ Estradiol	PNR	432	207	225 (COC)	6 cycles	Acceptability satisfaction sexual satis.	Quantitative; Questionnaire; Home interviews	10% of CVR users complained of difficulty with insertion, 20% of difficulty with removal, 43% worried with correct placement, 33% reported vaginal pain, and 10% reported having expelled it at some time. 17% of ring users and 7% of pill users considered their experiences “very good” but the general level of satisfaction with both methods was similar; women liked having control over use of the method, inserting and removing the ring at will for intercourse or washing. Increased libido reported by both CVR and pill users (50% users)
Faundes et al., 1981, Brazil, Dom. Rep. (47)	Levonorgestrel/ Estradiol	PNR	5,943	341	3,146 (COC) 2,456 (other methods)	10–23 cycles	Acceptability	Quantitative: Questionnaire	Field acceptance rate of the CVR (among other methods) Ranged in 4 sites from 2.9 to 12.5%.Ease of use was the most “liked” characteristic of the CVR.
Thiery et al., 1976, Belgium (16)	Medroxyprogesterone acetate	PR	14	14	2 arms different regimens	1 cycle	Acceptability	Quantitative: Questionnaire and diary	Not uncomfortable nor expelled or found displaced; no complaint on irritation or leukorrhea. *n* = 3 who had sexual intercourse reported “no interference with sexual relationship“.
Mishell 1972, USA (12)	Medroxyprogesterone acetate	PR	24	24	4 arms (initiation, ring)	6 cycles	Acceptability	Quantitative: Questionnaire	After initial fitting by a physician, the subjects removed and reinserted the devices themselves without difficulty. Both the subjects and their spouses stated the devices did not cause discomfort during coitus.
Weisberg et al., 1995; USA, Australia (17)	Norethindrone acetate/ Ethinyl Estradiol (EE)	PR	159	159	3 arms regimens	6 cycles	Acceptability satisfaction	Quantitative: including self-administered Questionnaire	Ring expulsion at low frequency in all 3 insertion groups. 69% rated the method as very good. 72% of women in Los Angeles and 62% in Sydney liked the ring much more than their most liked previous method. 92% in Sydney and 89% in Los Angeles would recommend the ring to others.
Schindler et al., 1993, Germany (42)	3-keto-desogestrel /EE	PNR	50	50	-	8 cycles	Acceptability	Quantitative: Questionnaire	For 96 and 93.5% of the women CVR was easy to insert and remove, respectively. Most partners (91%) felt the ring, but 96% did not consider it a problem.

*PR, prospective randomized; PNR, prospective non-randomized; CS, cross-sectional; UK, United Kingdom; USA, United States of America; CVR, contraceptive vaginal ring; COC, combined oral contraceptive; DMPA, depot-medroxy progesterone; IUD, intra uterine device*.

Overall satisfaction with NuvaRing^®^, measured on the 4–6-point Likert scale or using a dichotomous variable (Yes/No), ranged between 80 and 90%, with studies without a comparison group (other contraceptive method) more tending to report the highest satisfaction rates (Table 3). However, a high level of satisfaction (“being very satisfied”) varied across studies and ranged between 30 and 94% (the highest proportion reported in a study conducted in India) (14, 32, 34, 38). In Rwanda, general satisfaction with NuvaRing^®^ was high (over 80%) and concurred with qualitative findings and a ballot box (anonymous) survey at the end of the trial (26). In South Africa, more injection users (96.3%) significantly thought that this delivery mode was convenient to use compared to NuvaRing^®^ (83.1%; *p* = 0.0409) or COC (76.0%; *p* = 0.0034). Overall, the preferred contraceptive choice was injection, followed by the ring and lastly the pill (19). Willingness to recommend NuvaRing^®^ to others ranged from 60 to over 90% in the studies documenting satisfaction. NuvaRing^®^ can be used for 3 weeks, then removed for 1 week before reinsertion, or used for an extended period (or continuous use). In total, 3 studies documenting extended use of NuvaRing^®^ showed fair satisfaction/sexual satisfaction rates (11, 40, 49); although, in 1 study, satisfaction rates tended to be higher among women using shorter regimens (11). In a randomized study controlling for intermittent vs. continuous NuvaRing^®^ use in Rwanda, most women in both the groups reported similar acceptability and satisfaction and appreciated the absence of negative side effects (26).

A number of studies reported “sexual comfort” as whether NuvaRing^®^ was felt by partners (up to 30%), whether a number of partners found it bothersome or did mind (5–20%) (28, 31, 37, 45), objected its use (6%) (33), or did prefer the pill (5–30%) instead of CVR (18). A multicenter NuvaRing^®^ study in high-income settings pointed out that sexual comfort for the women who prematurely discontinued participation in the studies was only marginally lower than for those who completed them (33).

Sexual satisfaction while using NuvaRing^®^ was reported in a total in 16 studies, exclusively (*n* = 9) or with acceptability and general satisfaction (*n* = 7). Through the use of female sexual function indexes, scales, or diaries, these studies showed mixed sexual satisfaction results with NuvaRing^®^ use. In total, 2 studies (one without comparison group, another 1 comparing a new CVR Kirkos^®^ to NuvaRing^®^) conducted in Italy reported an improvement of all the variables between baseline and follow-up (40). In total, 2 prospective controlled studies conducted by Guida et al. in Italy reported improved overall and sexual relationship (“complicity”) among couples (27) and reduced anxiousness compared to COC users (55). Increased sexual desire (compared to COC or progestin-only pill) was reported among NuvaRing^®^ users in a small study in Belgium (22) and increased or unchanged sexual desire in another study comparing NuvaRing^®^ to low estrogen dose COC (13). In Rwanda, most women reported that ring use stimulated conversations with their partners about increased lubrication and sexual desire, but also about family planning and more general relationship topics. Most women (81%) reported at least once during ring use that the ring made sex feel better and this increased to 87% at the last study visit. Qualitative data confirmed this finding “this ring should be promoted as a sex enhancer” (26). The authors highlighted that Rwandan cultural norms around sexuality positively influenced the acceptance of the NuvaRing^®^. On the other hand, a randomized trial found that the McCoy Female Sexuality Questionnaire decreased significantly over treatment among COC and CVR users (10). A recent cross-sectional study reported significantly lower median values of female sexuality indexes in the CVR group compared to implant (53). In total, 2 studies found significantly decreased libido (3.3 vs. 0.8%) or mean female sexual function indexes with the ring compared to COC or patch users, respectively (21, 31).

Results with respect to continuation rates reported in our acceptability/satisfaction studies were mixed. Some NuvaRing^®^ studies showed a higher willingness to continue the use of the method (71% for CVR vs. 26.5% for skin patch users) (21) or a higher continuation rate (1% CVR users changed to pill or patch vs. 32–33% COC and skin patch users who changed to CVR) (21, 54). Other studies showed similar (high or low) continuation rates compared to COC [71 CVR vs. 75% COC (9, 23); 26 CVR vs. 29% COC (10)]. In total, 12 months continuation rates were lower (54%) for CVR users compared to 86% among long-acting reversible contraception [intra uterine device (IUD) and implant], 57% for depot-medroxyprogesterone acetate (DMPA), and 55% for COC, but higher than for skin patch users (49%) in a study conducted in the US. A recent study conducted among adolescents in South Africa showed that more NuvaRing^®^ users (24/116; 21%) significantly requested to change to another contraceptive option compared to injection (1/73; 1.4% *p* = 0.0002) and COC users (4/49; 8% *p* = 0.074) (19). Finally, 1 year treatment completion rates were higher (77%) with the shorter NuvaRing^®^ treatment regimens compared to 1-year extended regimen use (59%) (11).

Finally, opinions of male partner about NuvaRing^®^ were usually indirectly assessed by asking women about perception of the CVR of their partners (dimension and sexual comfort). Only 2 studies interviewed (qualitatively) the male partners themselves on perceptions and experiences with the ring (26, 30): In Kenya, experiences with CVR reflected a broader family planning (FP) paradigm: FP intentions and disclosure practices were influenced by partner support, socioeconomic factors, religion, cultural beliefs, and societal norms, including female sexuality (30). In Rwanda, finding from a limited number of interviews of male partners was in line with high acceptability and satisfaction reported by women (26).

#### Annovera™ and Progering^®^

Acceptability of Annovera™ was reported as high in 2 studies. Similar acceptability dimensions than in NuvaRing^®^ studies were used in an Annovera™ trial in Europe, USA, and Latin America and included in a theoretical framework presenting a pathway from acceptability to satisfaction then further to adherence and continuation (27). In the same study, satisfaction with Annovera™ was rated high (89%) and was associated to adherence and continuation (*p* < 0.001). Not feeling the ring while wearing it and experiencing no change or an increase in sexual pleasure and/or frequency was associated with higher odds of satisfaction (Table 3) (27). An earlier study showed an overall 1-year continuation rates at 73%. Medical conditions, mainly vaginal problems, personal reasons, and device loss or repeated expulsion, were the principal reasons given for study discontinuation (15).

Acceptability of the progesterone vaginal ring was rated high including ease to insert/remove/reinsert in African and Latin American settings and perceptions positively improved between the time the ring was first seen and the time it was used (36, 41) (Table 3). Perceptions of the size or texture of ring were reported of more importance than its color at baseline in African settings (36). In Latin America, 5–30% of women reported negative experiences (vaginal symptoms—excessive discharge or expulsion) (41), while in African settings expulsion reported rate was 5%. The study in sub-Saharan Africa included “family support” as an additional dimension of acceptability (36) and reported using a theoretical framework including other stakeholders such as healthcare providers, program managers, and policymakers, although the framework was not presented. In this study, data indicated that the CVR had limited to no effect on sexual behavior in the postpartum period (36).

#### Other Types of CVRs

Earlier studies on other types of CVRs reported on acceptability often referring to clinical features and tolerability. Vaginal symptoms, expulsions, and menstrual disturbances led to discontinuation among LNG 20 μg ring users (46, 51). Similarly, results from a qualitative study with the same ring conducted in the UK (parallel to the WHO randomized trial) showed that overall 7 of 27 women discontinued after a year and 4 of them for ring-related reasons (44). In a study conducted in the early 80s in Latin America with a LNG/estradiol ring, 43% of women reported being worried about correct ring placement/insertion (50) (Table 3).

## Discussion

Many studies using mostly quantitative structured questionnaires have documented acceptability and satisfaction of hormonal CVR, particularly NuvaRing^®^. The majority of these studies were conducted in high- or middle-income settings. Overall, CVR studies show that easiness to insert/remove/reinsert CVRs was high. Continuation rates, when reported, showed mixed results. Among NuvaRing^®^ studies, general satisfaction (being satisfied or very satisfied) was between 80 and 90%, although limited information was provided on actual experiences of women while using CVR; relationship attributes (such as couple communication and decision to use CVR); and contextual elements such as community perceptions of contraception and the CVR, gender/sexual norms, and experience.

Ease of insertion/removal/reinsertion of CVRs was reported in most included studies and rated high including in Latin American or African settings. Among the included studies, qualitative data on actual experiences of women while using the ring showed that initial worries related to CVR itself or its use, such as aspect, insertion, removal, and feeling the ring inside the vagina, improved over time (26, 30, 52), as it is also reported in 1 qualitative systematic review of CVR and 1 systematic review of vaginal rings (55, 56). Initial concerns sometimes required additional support from the provider or practice from the user (30, 55) or benefited of an adjustment period as reported among adolescents and younger users in the US (52). In addition, 2 other CVR studies have shown that “ease of use” was a major reason reported by participants for either selecting or using CVR in Spain (57, 58).

Perception of the ring of user was sparsely documented and data on ring expulsions were limited in the identified studies. However, when documented, ring-related reasons (slippage, expulsion, vaginal problems, or discomfort) contributed for a proportion of women to discontinuation of all the types of CVRs and confirmed findings from previous studies (55, 59). Expulsions and mechanical properties of the ring were included as a specific dimension in acceptability theoretical frameworks that were used in 2 included studies (26, 27) and in vaginal ring HIV prevention studies (8) and deserve to be further addressed in future studies.

Among NuvaRing^®^ studies, general satisfaction (being satisfied or very satisfied) was reported between 80 and 90%. However, as highlighted in our results, a comparison group (i.e., including the use of another contraceptive method or another regimen) was not present in about 40% of studies. Data triangulation between quantitative and qualitative data contributed to confirm or provide more information on satisfaction and factors, such as increased lubrication, leading to satisfaction and adherence (26, 36, 55).

Standard clinical trials in the field of CVRs mostly used structured questionnaires to assess acceptability, satisfaction, and/or sexual satisfaction. Mixed methods approaches combining quantitative and qualitative data collection were less commonly encountered. Clinical trial teams may be less familiar or reluctant to use qualitative approaches because this requires additional resources, time, and expertise over-and-above those required to carry out a clinical trial. Furthermore, qualitative study designs often use a purposive sampling strategy enrolling small numbers of participants, which is different from clinical trial designs based on representative sampling and statistical power calculations. Unlike CVR studies, HIV prevention vaginal rings studies often used mixed and qualitative methods and have documented acceptability of vaginal rings in low-income (high HIV prevalence) settings. These studies (8, 60) have highlighted the importance of using or incorporating qualitative study into clinical trial designs and the contribution of theoretical frameworks to better understand acceptability and satisfaction, as also shown in several studies of this study (25–27, 30, 36, 41, 44, 49, 52).

“Sexual comfort” usually referred to whether the ring was (reported) as felt either by women or partners during sexual intercourse or if the male partner “minded” the ring or its physical effects during intercourse (33). This issue raised concerns among less than a third of women in all the contexts studied. The regular set of acceptability dimensions used and information collected in NuvaRing^®^ acceptability studies did not include measures of frequency of sexual encounters or sexual satisfaction. Sexual satisfaction investigated most of the time in separate studies using female sexuality indexes or other similar measures that showed mixed results. According to a study by Sabatini and Cagliano, they pointed out that the analysis of adverse events revealed that disturbance of sexual intercourse was a crucial point for acceptability, compliance, and continuation (13). This is in line with other studies documenting the relationship between contraception and sexuality (61). Some authors believe that sexual side effects are the best predictors of discontinuation of oral contraceptives among heterosexual adult women (62). Actually, contraceptives can affect sexuality of women in a wide variety of ways beyond sexual functioning alone, for example, they can affect communication between sexual partners and empowerment of women (63). Interestingly, as qualitative data showed in Rwanda, enhanced communication of couples (for instance because of CVR use and potential increased lubrication) contributed to the acceptability of the NuvaRing^®^. The use of female sexual function indexes and aspects related to sexual relationship may help to improve our understanding of the relationships between contraception and sexuality including for CVRs.

When reported as it was not the focus of this study, willingness to continue CVR use or continuation rates showed mixed results compared to contraceptive pill users and skin patch. Some evidence suggests that long-acting contraceptives (implants or IUD) have higher continuation rates compared to short-acting contraceptives such as COC, but also CVR and skin patch. In South Africa, injectables showed the highest continuation rates and satisfaction.

Overall, limited information was provided on actual experiences of women using CVR and cultural context, which may affect CVR use (55). Further documenting actual experiences of women using the CVR and male partner opinions (including with respect to relationship and sexuality) can contribute to a better understanding of acceptability of and satisfaction with CVR (55). Awareness of the multiple contexts in experience of ring users and giving a strong voice to women with respect to their perception of contraceptive methods may inform the development and promotion approaches for CVR and more broadly vaginal rings (30, 64, 65).

This study has several limitations. First, given the lack of standardized definitions of acceptability and satisfaction, we may have missed articles documenting CVR acceptability or satisfaction that were not *explicitly* using this terminology and instead referred to continuation or adherence, which was not a specific outcome of interest in this study. Second, we could not always deduct from the methods sections of included studies whether interviews included open-ended questions. This may have led to under-recording of the use of semi-structured interviews. However in-depth qualitative techniques, such as IDIs or FGDs, were always clearly described in studies.

## Conclusion

Many studies using mostly quantitative structured questionnaires have documented acceptability and satisfaction of hormonal CVRs, particularly NuvaRing^®^. Despite the use of similar dimensions in a number of studies, there was a lack of standardized definitions of acceptability and satisfaction. Sexual satisfaction or pleasure was not typically included in acceptability dimensions and findings were not very informative in terms of actual experiences of women using CVRs and the cultural context that may affect sexuality and contribute to shape acceptability of CVRs. The use of mixed methods or qualitative approaches, including information on experiences of women using CVRs, relationship aspects, male partner opinions, and contextual sexual norms may lead to a better understanding of acceptability and satisfaction of CVRs. In addition, the use of theoretical acceptability frameworks highlighting the actual pathway from acceptability to satisfaction and adherence might also be useful.

## Author Contributions

TD, VJ, LB, and JW conceived and designed the study and wrote manuscript. TD, VJ, and LB conducted data-based searches. TD and VJ conducted data screening, selected the studies, performed data analysis, and presentation of results. TD conducted data extraction. All the authors provided input in manuscript writing and approved the final manuscript.

## Conflict of Interest

The authors declare that the research was conducted in the absence of any commercial or financial relationships that could be construed as a potential conflict of interest.

## Publisher's Note

All claims expressed in this article are solely those of the authors and do not necessarily represent those of their affiliated organizations, or those of the publisher, the editors and the reviewers. Any product that may be evaluated in this article, or claim that may be made by its manufacturer, is not guaranteed or endorsed by the publisher.
