# Supplementary material for: Acceptability and Satisfaction of Contraceptive Vaginal Rings in Clinical Studies: A Systematic Review and Narrative Synthesis
Source: Front Glob Womens Health. 2021 Dec 14;2:799963. doi: 10.3389/fgwh.2021.799963 (PMC8712726; doi:10.3389/fgwh.2021.799963)
Supplement: Supplementary file 2 [file Data_Sheet_2.pdf]

Supplementary Material S2 : List of excluded studies, CVR systematic review

	Authors names	Title	Year	Journal	Reasons for exclusion
1	Muller et al.	Safety, efficacy and quality of life of the novel vaginal contraceptive ring containing etonogestrel/ethinylestradiol 11.0/3.474 mg after 3 years of "real life" experience	2020	Eur Rev Med Pharmacol Sci	Review
2	Roy et al	Original Progesterone vaginal ring as a new contraceptive option for lactating mothers: Evidence from a multicenter non-randomized comparative clinical trial in India	2020	Contraception	Not documenting acceptability/satisfaction (continuation)
3	Undie Chi-Chi et al	Choosing and Using the Progesterone Vaginal Ring: Women's Lived Experiences in Three African Cities	2020	Patient Prefer Adherence	Report same study as RamaRaoa 2015 and taken into account
4	Lu et al	Qualitative Study of the Contraceptive Effect on Women's Sexual Experiences: Beyond Hormonal Effects	2019	Obstet Gynecol	Not documenting acceptability/satisfaction (Sexual experiences)
5	Murphy, DJ et al.	Post-use ring weight and residual drug content as potential objective measures of user adherence to a contraceptive progesterone vaginal ring	2019	Contraception	Not documenting acceptability/satisfaction (adherence)
6	McLellan-Lemal E et al.	Characteristics of women screened for a contraceptive intravaginal ring study in Kisumu, Kenya, 2014	2016		Hypothetical use
7	Das U et al.	Exploring vaginal ring acceptability for contraception and sexually transmissible infection protection in India: a qualitative research study	2015	Sexual Health	Hypothetical use
8	Diedrich JT et al.	Three-year continuation of reversible contraception	2015	American Journal of Obstetrics and Gynecology	Not documenting acceptability/satisfaction as such (Continuation rates only or pearl index)
9	Ishaku S et al..	Progesterone vaginal ring: results of an acceptability study in Nigeria	2015	Abuja, Nigeria, Population Council, 2015 Dec.	Report same study as the one included (RamaRaoa 2015)
10	Mane B et al..	[Progesterone vaginal ring: results of an acceptability study in Senegal]	2015	Dakar, Senegal, Population Council, 2015.	Report same study as RamaRaoa 2015
11	Morrow GK et al.	The Promise of Intravaginal Rings for Prevention: User Perceptions of Biomechanical Properties and Implications for Prevention Product Development	2015	PLoS One	Not documenting acceptability/satisfaction as such (mechanical properties)
12	Obare F et al.	Progesterone vaginal ring: results of an acceptability study in Kenya	2015	Dakar, Senegal, Population Council, 2015.	Report same study as RamaRaoa 2015
13	Rosen RK et al.	Meaning-making matters in product design: users' sensory perceptions and experience evaluations of long-acting vaginal gels and intravaginal rings	2015	Contraception	Not documenting explicitely acceptability/satisfaction

14	Schurmans C et al.	The ring plus project: safety and acceptability of vaginal rings that protect women from unintended pregnancy	2015	BMC Public Health	Protocol CVR study
15	Jost S et al.	Contraception's choice: Women's opinion, satisfaction and profile: Results of a French national survey of a representative sample of 5963 women	2014	Gynecologie Obstetrique & Fertilite	Not documenting explicitely acceptability/satisfaction
16	Lopez LM, et al.	Skin patch and vaginal ring versus combined oral contraceptives for contraception	2013	Cochrane Database of Systematic Reviews	Review
17	Qian JF	ARTICLE IN CHINESE [Clinical efficacy and safety of megestrol acetate vaginal ring]	2013	Zhonghua Yi Xue Za Zhi	Article in Chinese
18	Lete I et al.	Continuation Rate of Combined Hormonal Contraception: A Prospective Multicenter Study	2012	Journal of Womens Health	Not documenting explicitely acceptability/satisfaction (Continuation rates only)
19	Lopez LM et al.	Immediate start of hormonal contraceptives for contraception	2012	Cochrane Database of Systematic Reviews	Review
20	Mohamed et al.	Combined contraceptive ring versus combined oral contraceptive (30-µg ethinylestradiol and 3-mg drospirenone)	2011	Int Journal of Gynecology and Obstetrics	Not documenting acceptability/satisfaction
21	Terrell LR et al.	Acceptability of the Vaginal Contraceptive Ring Among Adolescent Women	2011	Journal of Pediatric and Adolescent Gynecology	Hypothetical use
22	Andelin et al 2010	Adherence and acceptability of the contraceptive ring compared with the pill among students: a randomized controlled trial	2010	Obst Gynec	Commentary
23	Bustillos-Alamilla E.	[Combined hormonal contraception in cycles artificially extended]	2010	Ginecologia y Obstetricia de Mexico	Not found
24	Lopez LM et al.	Skin patch and vaginal ring versus combined oral contraceptives for contraception	2010	Cochrane Database of Systematic Reviews	Review
25	Fait T.	[Vaginal combined contraception NuvaRing in the clinical practice in the Czech Republic]	2009		Article in Czek
26	Raine TR, et al.	Attitudes Toward the Vaginal Ring and Transdermal Patch Among Adolescents and Young Women	2009	Journal of Adolescent Health	Not documenting acceptability/satisfaction
27	Colwell HH et al.	The ORTHO BC-SAT--a satisfaction questionnaire for women using hormonal contraceptives	2006		Validation study
28	Mathias SD, et al.	ORTHO birth control satisfaction assessment tool: assessing sensitivity to change and predictors of satisfaction	2006	Contraception	Validation study

29	Victor I & Fink RA.	Comparing patient telephone callback rates for different hormonal birth control delivery systems	2006		Not found
30	Miller L, verhoeven C & in't Hout J.	Extended Regimens of the Contraceptive Vaginal Ring. A Randomized Trial	2005	Obstetrics & Gynecology	Not documenting explicitely acceptability/satisfaction
31	Oddsson K	Efficacy and safety of a contraceptive vaginal ring (NuvaRing) compared with a combined oral contraceptive: a 1-year randomized trial	2005	Contraception	Not documenting explicitely acceptability/satisfaction
32	Weisberg E, Brache V, Alvarez F, et al.	Clinical performance and menstrual bleeding patterns with three dosage combinations of a Nestorone (R) progestogen/ethinyl estradiol contraceptive vaginal ring used on a bleeding-signaled regimen	2005	Contraception	Not documenting explicitely acceptability/satisfaction
33	Novak A, de la Loge C , & Abetz L.	Development and validation of an acceptability and satisfaction questionnaire for a contraceptive vaginal ring, NuvaRing	2004	PharmacoEconomics	Validation study
34	Sivin I et al.	Two-year performance of a Nestorone (R)-releasing contraceptive implant: a three-center study of 300 women	2004	Contraception	Not documenting explicitely acceptability/satisfaction
35	Book	New Frontiers in Contraceptive Research: A Blueprint for action.	2003		Full report; BOOK
36	Coren C.	Contraceptive ring found safe, effective: most users would recommend it	2003	Perspectives On Sexual and Reproductive Health	No original data/ Not documenting explicitely acceptability/satisfaction
37	Sarkar NN.	Steroidal contraceptive vaginal rings	2003	Int Journal of Clinical Practice	No original data/Opinion paper
38	Dieben TOM, Roumen FJME & Apter D.	Efficacy, cycle control, and user acceptability of a novel combined contraceptive vaginal ring	2002	Obstetrics and Gynecology	Same study as the one included in the review (Novack 2003°
39	Roumen F.	Contraceptive efficacy and tolerability with a novel combined contraceptive vaginal ring, NuvaRing	2002	European Journal of Contraception and Reproductive Health Care	Not documenting acceptability/satisfaction as such (Continuation rates only or pearl index)
40	Szarewski A.	High acceptability and satisfaction with NuvaRing use	2002	European Journal of Contraception and Reproductive Health Care	Same study as the one included in the review (Novack 2003°
41	Massai R et al.	Preregistration study on the safety and contraceptive efficacy of a progesterone-releasing vaginal ring in Chilean nursing women	1999	Contraception	Not documenting acceptability/satisfaction (pharmacokinetic, safety, efficacy)

42	Chen JH et al.	The comparative trial of TCu 380A IUD and progesterone-releasing vaginal ring used by lactating women	1998	Contraception	Article in Chinese
43	Faundes A.	Simplifying NFP: preliminary report of a pilot study of the 'collar' method in Brazil	1997	Advances in Contraception	Not hormonal CVR
44	Weisberg E et al.	Effect of different insertion regimens on side effects with a combination contraceptive vaginal ring	1997	Contraception	Report same study (Weisberg 1995)
45	Ballagh SA.	Contraceptive vaginal ring releasing norethindrone acetate and ethinyl estradiol	1994	Contraception	Not documenting acceptability/satisfaction as such
46	Olsson SE	Contraception with a vaginal ring releasing 3-keto desogestrel and ethinylestradiol	1990	Contraception	Not found
47	World Health Organization (HRP)	Microdose intravaginal levonorgestrel contraception: a multicentre clinical trial. II. Expulsions and removals	1990	Contraception	Not documenting acceptability/satisfaction (mechanical properties)
48	Elstein M & Nuttall ID	The progestagen releasing vaginal ring	1985	J Obstet Gynaecol (Lahore)	Report same study (Spencer 1986)
49	Gao J.	Clinical investigation of low dose levonorgestrel releasing vaginal ring	1984		[Unpublished] [1985] & report same study (Koetsawang 1990)
50	Hardy EE et al.	The hormonal ring. A new contraceptive gains acceptance	1984	IDRC Rep	Report same study (Hardy 1983)
